# Wortmannin-induced vacuole fusion enhances amyloplast dynamics in Arabidopsis *zigzag1* hypocotyls

**DOI:** 10.1093/jxb/erw418

**Published:** 2016-11-05

**Authors:** Ashley Ann Alvarez, Sang Won Han, Masatsugu Toyota, Carla Brillada, Jiameng Zheng, Simon Gilroy, Marcela Rojas-Pierce

**Affiliations:** ^1^Department of Plant and Microbial Biology, North Carolina State University, Raleigh, NC, USA; ^2^Japan Science and Technology Agency (JST), Precursory Research for Embryonic Science and Technology (PRESTO), Saitama, Japan; ^3^Department of Botany, University of Wisconsin, Madison, WI, USA; ^4^Department of Biochemistry and Molecular Biology, Saitama University, Saitama, Japan

**Keywords:** Amyloplasts, cytoskeleton, gravitropism, SNARE, vacuole, wortmannin.

## Abstract

Gravitropism in Arabidopsis shoots depends on the sedimentation of amyloplasts in the endodermis, and a complex interplay between the vacuole and F-actin. Gravity response is inhibited in *zigzag-1* (*zig-1*), a mutant allele of *VTI11*, which encodes a SNARE protein involved in vacuole fusion. *zig-1* seedlings have fragmented vacuoles that fuse after treatment with wortmannin, an inhibitor of phosphatidylinositol 3-kinase, and underscore a role of phosphoinositides in vacuole fusion. Using live-cell imaging with a vertical stage microscope, we determined that young endodermal cells below the apical hook that are smaller than 70 μm in length are the graviperceptive cells in dark-grown hypocotyls. This result was confirmed by local wortmannin application to the top of *zig-1* hypocotyls, which enhanced shoot gravitropism in *zig-1* mutants. Live-cell imaging of *zig-1* hypocotyl endodermal cells indicated that amyloplasts are trapped between juxtaposed vacuoles and their movement is severely restricted. Wortmannin-induced fusion of vacuoles in *zig-1* seedlings increased the formation of transvacuolar strands, enhanced amyloplast sedimentation and partially suppressed the agravitropic phenotype of *zig-1* seedlings. Hypergravity conditions at 10 *g* were not sufficient to displace amyloplasts in *zig-1*, suggesting the existence of a physical tether between the vacuole and amyloplasts. Our results overall suggest that vacuole membrane remodeling may be involved in regulating the association of vacuoles and amyloplasts during graviperception.

## Highlight

The highly restricted movement of amyloplasts in graviperceptive cells of *zig-1* hypocotyls is overcome by wortmannin treatment, suggesting a physical association between amyloplasts and the vacuole.

## Introduction

Plants have the capacity to sense and respond to the direction of gravity by precise modulation of growth patterns in roots and shoots (Hashiguchi *et al.*, 2013). Gravitropism involves mechanisms of perception, signal transduction and auxin-directed differential growth. According to the starch-statolith hypothesis, sedimentation of amyloplasts in response to gravity stimuli is critical for gravity sensing (Masson *et al.*, 2002). Gravity perception in Arabidopsis shoots depends on the ability to sense the position of starch-filled amyloplasts, called statoliths, within shoot endodermal cells, or statocytes (Fukaki *et al.*, 1998). This is supported by evidence that (i) mutants lacking an endodermis display agravitropic shoots (Fukaki *et al.*, 1998), (ii) starchless mutants or mutants that lack amyloplasts show reduced gravitropism (Kiss *et al.*, 1997; Weise and Kiss, 1999; Fujihira *et al.*, 2000), (iii) hypergravity restored gravitropic sensitivity in starch-deficient mutants (Fitzelle and Kiss, 2001), and (iv) there is significant movement (sedimentation) of amyloplasts following gravistimulation (Saito *et al.*, 2005; Hashiguchi *et al.*, 2014). The sedimentation of amyloplasts in shoot statocytes is thought to trigger downstream signaling events, including changes in the transport of and response to auxin (Rakusova *et al.*, 2011). The early events of signal transduction for gravitropism remain largely unknown, although several genes have been suggested to function in its early phases (Blancaflor and Masson, 2003).

The current model of gravity sensing implicates the plant vacuole in the shoot gravity response (Morita *et al.*, 2002; Yano *et al.*, 2003; Hashiguchi *et al.*, 2013). Shoot endodermal cells contain a large central vacuole that occupies most of the cell volume, and its role in gravity sensing is well established. Mutants with vacuolar defects, including mutant alleles of *SHOOT GRAVITROPISM6* (*SGR6*), *VTI11/ZIGZAG-1* (*ZIG-1*)/*SGR4*, *SYNTAXIN OF PLANTS22* (*SYP22*)/*SGR3* and *KATAMARI2* (*KAM2*) were identified from screens for agravitropic shoots in Arabidopsis (Fukaki *et al.*, 1996*b*; Yamauchi *et al.*, 1997; Kato *et al.*, 2002; Morita *et al.*, 2002; Yano *et al.*, 2003; Saito *et al.*, 2005; Hashiguchi *et al.*, 2014). A common phenotype in these mutants was the abnormal localization of amyloplasts in relation to the vacuole or abnormal vacuole morphology. In the case of *zig-1* mutants, the movement of amyloplasts in the endodermis of the inflorescence stem was greatly reduced (Yano *et al.*, 2003). Both VTI11 and SYP22 are SNARE (soluble N-ethylmaleimide-sensitive factor attachment protein receptor) proteins that have important roles in membrane fusion at the vacuole, including homotypic vacuole fusion (Chen and Scheller, 2001; Wickner, 2010; Zheng *et al.*, 2014*b*). Two mutant alleles of *VTI11*, *zig-1* and *impaired traffic to tonoplast3* (*itt3*), have multiple vacuoles per cell (Zheng *et al.*, 2014*a*,b). Treatment of *itt3* and *zig-1* with wortmannin (Wm), an inhibitor of phosphatidylinositol 3-kinase (PI3-kinase), resulted in vacuole fusion, and enhanced hypocotyl gravitropism (Zheng *et al.*, 2014*b*). This result is consistent with fragmented vacuoles being responsible for the agravitropic phenotype of *zig-1*. Two other proteins associated with endomembranes were implicated in gravitropism. KAT2 is a membrane-associated protein (Tamura *et al.*, 2007), and SGR6 is a HEAT-domain containing protein involved in vacuole membrane remodeling (Hashiguchi *et al.*, 2014). The roles of KAT2 or SGR6 in gravitropism are still unknown.

A tight association between amyloplasts and the large central vacuole has been described in inflorescence endodermal cells. In resting cells, amyloplasts are found at the bottom of the cell, where they undergo saltatory movements and are almost completely surrounded by the vacuole membrane (Morita *et al.*, 2002; Yano *et al.*, 2003; Morita, 2010). Amyloplast sedimentation that results from changes in the gravity vector occurs inside transvacuolar strands (TVSs), and the amyloplasts remain tightly associated with the vacuolar membrane during this time (Saito *et al.*, 2005; Moirta *et al.*, 2012). TVSs may allow movement of organelles, and their formation requires actin filaments (Gao *et al.*, 2005; Leitz *et al.*, 2009; Moirta *et al.*, 2012). It is unclear if the F-actin inside TVSs is required for amyloplast association with the vacuole, sedimentation, or mechanisms of gravity perception (Morita, 2010; Blancaflor, 2013), but F-actin cables have been shown to associate with sedimenting amyloplasts inside TVSs (Saito *et al.*, 2005). However, the tight association between vacuole and amyloplasts was not disrupted by lantrunculin-B in wild-type inflorescences (Saito *et al.*, 2005). Overall, the mechanisms by which amyloplasts are so closely attached to vacuolar membranes, including TVSs, and the role of this interaction for gravitropism have not been elucidated. Interestingly, *kat2* mutants, which are agravitropic in shoots, develop fewer transvacuolar strands in leaves and petioles (Tamura *et al.*, 2007), but it is not known if this is the case for shoot endodermal cells. Even though a clear role for the vacuole in gravity perception is well established, the molecular mechanisms for vacuolar control or perception of amyloplast sedimentation has not been identified.

In addition to the vacuole, important roles for actin filaments have been reported, but their role during shoot gravitropic perception remains unclear (Blancaflor, 2013). Actin depolymerizing drugs have been reported to enhance gravity response in hypocotyls (Yamamoto and Kiss, 2002; Yamamoto *et al.*, 2002), reduce amyloplast sedimentation in endodermal cells (Palmieri and Kiss, 2005) or have no effect in experiments with live cells (Saito *et al.*, 2005). An important role of the actin cytoskeleton was demonstrated by the identification of the *shoot gravitropism9* (*sgr9*) mutant, which shows reduced amyloplast sedimentation due to increased interactions between amyloplasts and F-actin (Nakamura *et al.*, 2011). *SGR9* encodes an E3 ligase that may disrupt the attachment of amyloplasts to F-actin during saltatory movements and as a result promote amyloplast sedimentation (Nakamura *et al.*, 2011). The difficulty of establishing a role of F-actin in gravity perception may be due to the complexity of interactions that it may have with a multitude of cellular components, including amyloplasts.

Here we used live-cell imaging on a vertical stage microscope to demonstrate the role of the large central vacuole in shoot gravitropism in seedlings. Our results identified the upper hypocotyl endodermis as the gravisensing tissue and highlight a novel tight interaction between the vacuolar membrane and amyloplasts. The fragmented vacuoles in endodermal cells of *zig-1* hypocotyls restrict amyloplast movement, contributing to the agravitropic phenotype of this mutant. We show that application of Wm to *zig-1* enhances their gravitropic response, restores amyloplast sedimentation and enhances the formation of TVSs. Additionally, we show that the tight association between vacuoles and amyloplasts observed in *zig-1* hypocotyls involves neither F-actin nor microtubules.

## Materials and methods

### Plant material and growth conditions

The *zig-1* mutant line was previously described (Kato *et al.*, 2002). The parental and *itt3* lines contain the green fluorescent protein (GFP)-TIP2;1 and the mCherry-HDEL markers (Zheng *et al.*, 2014*b*), and marker lines expressing the fluorescent protein fusions VHA-a1:GFP (Dettmer *et al.*, 2006), ABD2-GFP (Wang *et al.*, 2004) and TUA6-GFP (Ueda *et al.*, 1999) were described before. Seeds were surface sterilized and incubated at 4 °C in the dark for 2–5 d. Seeds were sown on Arabidopsis growth medium (AGM; 0.5× Murashige and Skoog medium, 4 g l^–1^ GelRite, 1% (w/v) sucrose) and transferred to a 22 °C growth chamber with 16 h/8 h day/night photoperiod. For dark grown experiments, plants were incubated in light for 8 h to stimulate germination prior to growth in the dark.

### Plasmid construction and plant transformations

The *pSCR::TIP1;1-GFP* construct was generated by amplifying a 2 kb fragment corresponding to the *SCARECROW* promoter (pSCR2.0) from pENTR 5′pSCR2.0 (Levesque *et al.*, 2006; Sozzani *et al.*, 2010) using primers 5′-AAGCTTTGCCAAA CAGATATTTGCATTTGGGCTATG-3′ and 5′-CTCGAGTAGG AGATTGAAGGGTTGTT-3′. The pSCR2.0 PCR fragment was cloned into VAC-GK (Nelson *et al.*, 2007) by restriction enzyme and ligation with *Hin*dIII and *Xho*I to generate *pSCR::TIP1;1-GFP*. The resulting plasmid was then transformed into *zig-1* or Col-0 WT by the floral dip method (Clough and Bent, 1998).

### Chemical stocks and treatments

All chemical treatments used 4-d-old seedlings unless otherwise specified. Stock solutions of 3.3 mM wortmannin (Sigma-Aldrich), 200 μM latrunculin B (Lat-B; Sigma-Aldrich), and 2 mM oryzalin (Sigma-Aldrich) were made in 100% (v/v) dimethyl sulfoxide (DMSO) and diluted to working concentrations in liquid AGM as 1% (v/v) DMSO, 33 μM Wm, 2 μM Lat-B and 20 μM oryzalin. Vacuole fusion assays were carried out by incubating seedlings in either DMSO or Wm for 90 min to 2 h before rinsing in diH_2_O and imaging.

Lugol solution was purchased from Sigma-Aldrich (62650). Staining was carried out by submerging Col-0 WT seedlings in solution for 20 min, while maintaining their growth orientation. Next, they were washed in diH_2_O for 10 min before they were mounted on microscope slides.

### Microscopy

Imaging in a vertical stage was carried out with a Leica DM5000 compound microscope equipped with a Leica DFC365 FX camera, and a Leica ×40/1.0 NA water objective was used. The microscope was outfitted with a custom-made 90° InverterScope objective inverter (LSM Technologies) between the objective turret and the ×40 objective and a custom-made vertical stage (LSM Technologies) that included a manual rotation stage (Thor Labs). Bright-field imaging was accomplished by placing an aspheric condenser lens (cat. no. ACL1210-A, Thor Labs) inside the rotation stage ~10 mm behind the sample, and an adaptor was made by 3-D printing to attach the end of a light guide to the back of the vertical stage. The adaptor included a slit to slide a blocking sheet or a green filter (see Supplementary Fig. S1 at *JXB* online). All imaging was done with the green filter inside the slit. Etiolated seedlings were affixed to slides using a very fine layer of Hollister Medical Adhesive (Behera and Kudla, 2013). A 1.0 mm silicon isolator (cat. no. CWS-13R-1.0, Grace BioLabs) and a coverslip were used to create a chamber filled with water, and Immersol 518 F (Carl Zeiss) was used as the immersion oil for the water objective.

A Zeiss LSM 710 confocal microscope with a ×40 water objective (1.1 NA) or ×20 objective (0.8 NA) was used for confocal microscopy. The excitation/emission wavelengths during acquisition were 488 nm/492–557 nm for GFP, and 516 nm/582–670 nm for mCherry. TVSs were counted as previously described (Han *et al.*, 2015). Briefly, hypocotyls were imaged in 600 μm thick optical sections using a ×20 (0.8 NA) objective to capture the volume of entire cells. Vacuolar membrane folds were counted as TVSs if they were formed by a double membrane, even if they did not transverse the entire vacuole in the *XY* plane.

A centrifuge microscope was used for hypergravity experiments as previously described (Toyota *et al.*, 2013*a*,b). Four-day-old etiolated seedlings were mounted in a chamber to fix the specimen on the centrifuge microscope before rotation; 10 *g* was applied after 10–20s of imaging and held continuously for 60s.

### Quantification of organelle movement

Tracking amyloplast movement was performed with the MTrackJ plugin of ImageJ. Amyloplast movement was quantified capturing time-lapse images for 3 min at 10 s intervals, but only amyloplasts that could be traced throughout all frames were scored. Seedlings were 4 d old for all experiments. The *x*, *y* coordinates of each amyloplast were recorded from each image. Then, the change in position in the *x* (Δ*x*) and *y* (Δ*y*) axes were determined and plotted in a coordinate graph. A stationary reference point such as the corner of a cell was used to correct for stage drift.

Tracking of amyloplasts during hypergravity experiments was carried out with G-Track spot-tracking software (G-Angstrom, Japan) as described (Toyota *et al.*, 2013*a*).

### Gravitropism assay and hypocotyl curvature

Col-0 WT and *zig-1* were sown on AGM, wrapped in foil to simulate dark, and oriented vertically for 3 d. All subsequent transfers took place in a dark room using a green safe light. Wm treatment was carried out by first aligning seedlings to the first gravity vector (*g*1) before placing an AGM dot containing either 1% (v/v) DMSO or 33 μM Wm on the top or bottom of the seedlings. For cytoskeleton inhibitor treatments, seedlings were transferred to fresh plates containing DMSO, 2 μM Lat-B or 20 μM oryzalin, and aligned to *g*1. Plates were wrapped in foil after transfers, rotated 90°, and scanned after another 20 h in the dark. ImageJ was used to measure hypocotyl curvature as the angle between the top of the hypocotyl and *g*1. All experiments were repeated at least three times.

## Results

### Statocytes in etiolated hypocotyls are restricted to young cells below the apical hook

**Fig. 1.**
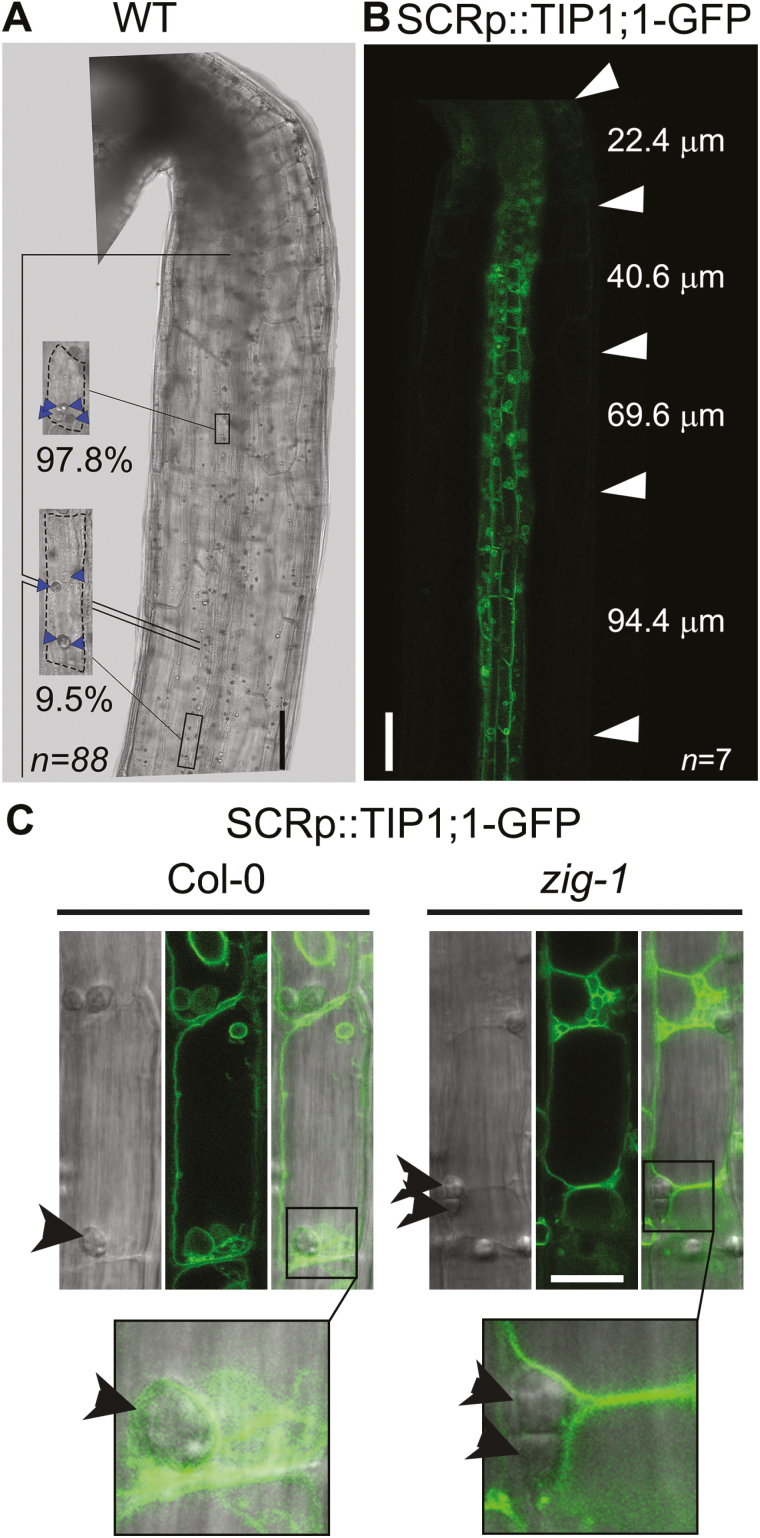
Graviperceptive cells are located just below the shoot apex. (A) Endodermal cells below the apical hook contain sedimented amyloplasts. Col-0 WT seedlings were stained with Lugol solution and imaged on a compound microscope while maintaining a vertical orientation. The insets show representative cells from the region just below the apical hook (top) or a region towards the middle of the hypocotyl (bottom). Percentages under the insets represent the proportion of amyloplasts found at the bottom of cells in each region of the hypocotyl. *n*=88 amyloplasts from 23 cells and three seedlings. Arrowheads indicate the position of amyloplasts. Scale bar: 100 μm. (B) Increase of cell length along the hypocotyl endodermis. Dark-grown Col-0 WT seedling expressing *pSCR::TIP1;1-GFP* were imaged by confocal microscopy. Average length of cells between the arrowhead marks is shown. Cell length data also include seedlings stained with Lugol solution and imaged by bright-field. *n*=7 seedlings. Scale bar: 100 μm. (C) Amyloplasts get trapped between vacuoles in *zig-1* endodermis. Endodermal cells near the shoot meristem of Col-0 WT (Col-0) and *zig-1* seedlings expressing *pSCR::TIP1;1-GFP* were imaged by bright-field and confocal microscopy. Arrowheads mark the position of amyloplasts. Insets show the association between tonoplast and amyloplast. Scale bar: 20 μm.

Amyloplast sedimentation in shoots has been well characterized by live-cell microscopy in inflorescence stems (Morita *et al.*, 2002; Saito *et al.*, 2005; Toyota *et al.*, 2013*b*) but not in seedlings. In order to characterize mechanisms of gravity perception in seedlings, we first quantified amyloplast sedimentation in wild-type (WT) hypocotyl endodermal cells by Lugol staining (Fig. 1A). When cells from the upper portion of the hypocotyl were imaged, 97.8% of the amyloplasts were found sedimented in the direction of gravity. However, only 9.5% of amyloplasts were observed at the bottom of the cell when cells were imaged further down the hypocotyl. This result suggests that amyloplast sedimentation may be restricted to endodermal cells just below the apical hook.

We measured cell length along the upper hypocotyl endodermis to better understand the potential transition between statocytes and non-statocytes. Cell length was estimated from bright-field images of Lugol-stained WT, as well as confocal images of a GFP-tagged vacuolar marker exclusively expressed in the endodermis (pSCR::TIP-1;1-GFP, Fig. 1B). Cell length varied from 23 ± 3.69 to 94 ± 0.82 μm in this region when measured from the apical hook to ~1 mm towards the root, but on average, only cells with length <70 μm showed sedimented amyloplasts. Using amyloplast sedimentation as a marker for the ability to perceive gravity, the small cells at the top of the hypocotyl are more likely to correspond to the shoot statocytes. Given this result, all subsequent experiments described below were conducted with endodermal cells between 30 and 70 μm in length within this region of the hypocotyl.

Amyloplasts in graviperceptive cells have been shown to be closely associated with the vacuole membrane (Morita *et al.*, 2002), and mutants in the SNARE protein VTI11 have abnormal vacuole morphology (Zheng *et al.*, 2014*b*) and agravitropic phenotypes (Kato *et al.*, 2002). The vacuole was visualized in WT and *zig-1* using the pSCR::TIP1;1-GFP marker in order to determine the relationship between the vacuole and amyloplast in seedlings. As expected, a large central vacuole occupies most of the volume of WT endodermal cells and the tonoplast surrounds the sedimented amyloplasts (Fig. 1C). In contrast, *zig-1* displays a fragmented vacuole phenotype in shoot endodermal cells, as previously described for other tissues (Zheng *et al.*, 2014*b*). It is important to note that amyloplasts do not sediment in *zig-1* mutants and appear to be trapped at the junction between two or more adjacent vacuoles (Fig. 1C). Furthermore, and similar to *zig-1* inflorescences (Morita *et al.*, 2002; Saito *et al.*, 2005), the tonoplast does not tightly surround the amyloplasts in *zig-1* hypocotyls. These results indicate that the vacuole phenotype of *zig-1* impinges on the distribution of amyloplasts in hypocotyl endodermal cells and likely prevents their sedimentation.

### The dynamic behavior of endodermal amyloplasts is restricted in *zig-1*

**Fig. 2.**
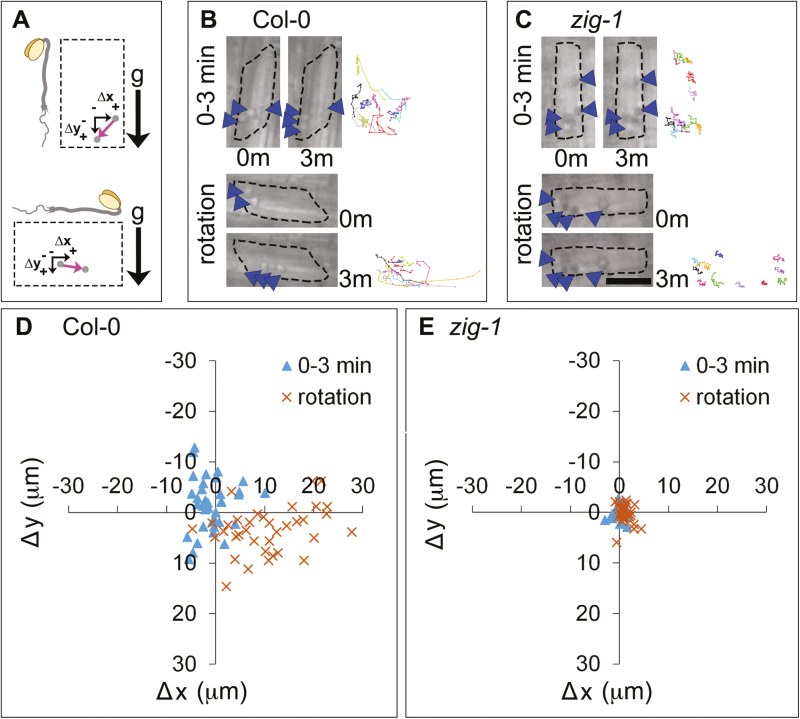
Amyloplast dynamics in hypocotyl endodermal cells before and after gravity reorientation. (A) Schematic representation of the rotation experiment. The orientation of the seedling with respect to gravity (g) before and after rotation is shown. Displacement of an amyloplast (gray circle) during a 3 min time lapse is shown with a pink arrow, and is expressed as a change in the horizontal position (Δ*x*) and the vertical position (Δ*y*). Positive values correspond to right and down and negative values correspond to left and up. (B, C) Amyloplast movements before and after rotation. A representative cell from the endodermis of Col-0 WT (B) or *zig-1* (C) is shown before (0–3 min) or after (rotation) a 90° rotation. Images correspond to the amyloplast positions at the start (0m) or the end (3m) of a 3 min time lapse. Tracks of representative amyloplasts are shown as different colored lines at the right of each panel. Arrowheads indicate one to two amyloplasts. Scale bar: 15 μm. (D, E) Quantification of amyloplast movements in WT and *zig-1* before and after rotation. Coordinate graph showing amyloplast displacement between 0 and 3 min in Col-0 WT (D) or *zig-1* (E). Time-lapse experiments were carried out either before (blue triangles) or after (orange crosses) a 90° rotation. *n*=73 amyloplasts from four seedlings of Col-0; *n*=65 amyloplasts from four seedlings of *zig-1*.

A novel set-up for live-cell imaging on a vertical microscope was developed to identify mechanisms of vacuolar membrane dynamics involved in gravity perception or response (see Supplementary Fig. S1). Seedlings were grown vertically for 4 d before being mounted in a slide chamber and equilibrated for 5 min. Sequential images were acquired at 10 s intervals for 3 min (Saito *et al.*, 2005) in the vertical stage (Fig. 2A). Most WT amyloplasts were detected at the bottom of the cell between 0 and 3 min (Fig. 2B, 0–3 min), and organelle tracking indicates that they exhibited dynamic saltatory movements (Fig. 2B, 0–3 min). In contrast, *zig-1* amyloplasts were found more widely distributed in the cell between 0 and 3 min, and they were severely limited in their movement as shown by their comparatively static tracks (Fig. 2C). Next, seedlings were rotated 90° and imaged immediately for the following 3 min (Fig. 2A). Amyloplasts from WT sedimented in the direction of gravity after reorientation as shown by the organelle tracks between 0 and 3 min after rotation (Fig. 2B, rotation). In contrast, amyloplasts from *zig-1* continued to be much more limited in their movement even after a reorientation (Fig. 2C, rotation). These results, and those from inflorescences (Saito *et al.*, 2005), support the role of the central vacuole in shoot gravity sensing.

Quantification of amyloplast movement indicated that their movement in WT before rotation occurred in all directions for up to 10 μm (Fig. 2D, E). Following a 90° reorientation, most amyloplasts translocated to the new bottom of the cell (lower right quadrant of the graph) for up to 30 μm. On the other hand, amyloplast movement in *zig-1* was confined to <5 μm in all directions before reorientation, and there was little change in their position after a 90° reorientation (Fig. 2E). These results indicate that the fragmented vacuoles may impair movement of these organelles in *zig-1*.

**Fig. 3.**
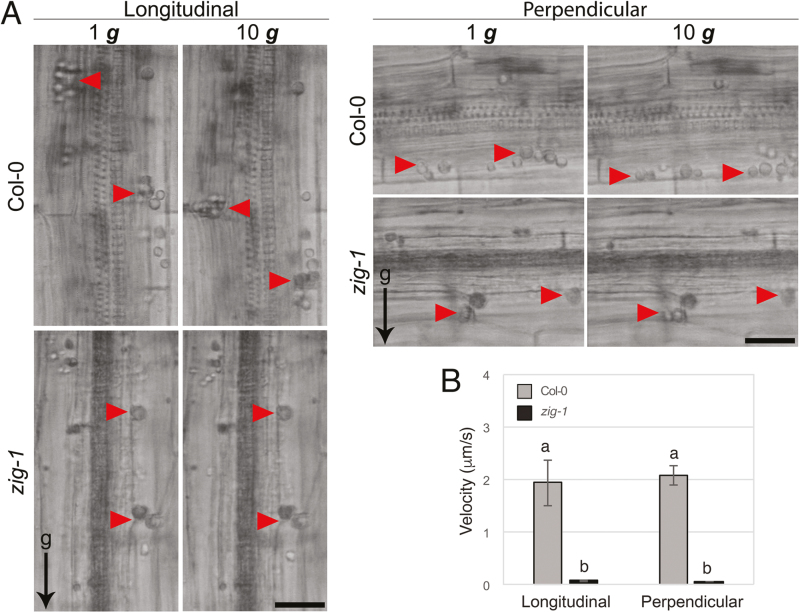
Hypergravity does not displace amyloplasts in the *zig-1* mutant. (A) Hypergravity (10 *g*) was applied either longitudinally or perpendicularly to 4-d-old dark grown WT and *zig-1* seedlings. Amyloplast localization was imaged by bright-field. Amyloplasts in WT relocate to the gravity associated bottom of the cell within 1 min of hypergravity; however, amyloplast movement in *zig-1* is much more restricted. Scale bar: 20 μm. (B) The velocity of amyloplast movement in Col-0 and zig-1 was analysed under the 10 *g* conditions. Data are shown as means and error bars represent standard error. Different letters denote significant differences among all four data (*P*<0.05, one-way ANOVA followed by the Tukey’s multiple comparison test). *n*=9–17 amyloplasts. (This figure is available in color at *JXB* online.)

Reduced amyloplast sedimentation in *zig-1* hypocotyls could be due to the physical impediments created by the fragmented vacuoles. To address this question, we utilized hypergravity conditions to increase sedimentary movement and analysed amyloplast behavior using a centrifuge microscope (Toyota *et al.*, 2013*a*,b). Two types of gravistimulation were used at 10 *g*, one applied longitudinally (along the shoot–root longitudinal axis) and a second applied perpendicularly, similar to a 90° reorientation assay. Upon application of hypergravity in either direction, amyloplasts in WT moved and reached the edge of the cell within 60 s (Fig. 3A). Surprisingly, when 10 *g* was applied in either direction in *zig-1*, amyloplast movements were highly restricted (Fig. 3A), indicating a potential tight association between amyloplasts and the tonoplast itself. Measurements of amyloplast velocity indicated that these differences are significant (Fig. 3B). Collectively, these results suggest that failure of amyloplasts to sediment in *zig-1* is due to the abnormal vacuoles and a potential physical interaction between vacuoles and amyloplasts.

### Wortmannin-induced vacuolar membrane fusion releases endodermal amyloplasts in *zig-1*

**Fig. 4.**
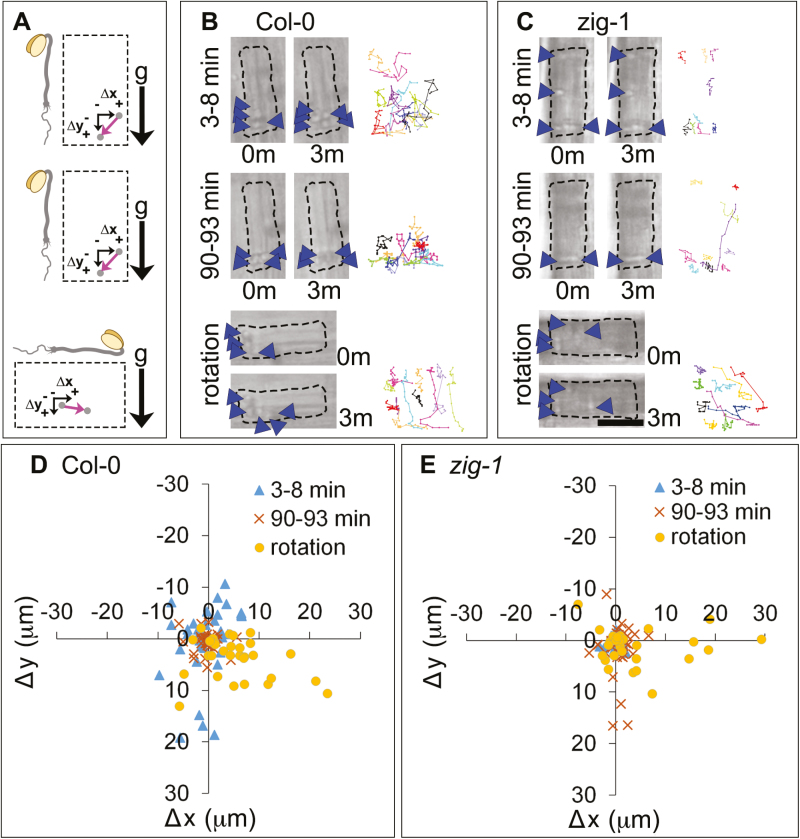
Wortmannin treatment enhances amyloplast dynamics in *zig-1* hypocotyls. (A) Representation of the rotation experiment showing the orientation of the seedling with respect to gravity throughout the experiment. Displacement of an amyloplast (grey circle) during a 3 min time lapse is shown with a pink arrow, and is expressed as a change in the horizontal (Δ*x*) and the vertical (Δ*y*) position. Positive values correspond to displacement to the right and down while negative values correspond to displacement towards the left and up. Seedlings were incubated vertically in the presence of 33 μM Wm and imaged immediately for 3 min (top, 3–8 min). Another 3 min time lapse was captured after 90 min of Wm treatment (middle, 90–93 min), and finally, seedlings were rotated 90° and a 3 min time lapse was captured immediately (bottom, rotation). (B, C) Amyloplast movements after a Wm treatment. A representative cell from the endodermis of Col-0 WT (B) or *zig-1* (C) was imaged immediately after Wm treatment (3–8 min), 90 min after Wm treatment (90–93min) or 90 min after Wm treatment and a 90° rotation (rotation). Images correspond to the amyloplast positions at the start (0m) or the end (3m) of a 3 min time lapse. Tracks of representative amyloplasts are shown as different colored lines at the right of each panel. Arrowheads indicate one to two amyloplasts. Scale bar: 15 μm. (D, E) Quantification of amyloplast movements in WT and *zig-1* after Wm treatment. Coordinate graph showing amyloplast displacement between 0 and 3 min of each time lapse in Col-0 WT (D) or *zig-1* (E). Time lapse experiments were carried out within 3–8 min of Wm treatment (blue triangle), after 90 min of Wm treatment before rotation (orange crosses), or after 93 min of Wm treatment followed by a 90° rotation (yellow circles). *n*=110 amyloplasts from four seedlings of Col-0; *n*=98 amyloplasts from four seedlings of *zig-1*.

Wm was used to test the effect of vacuole fusion on amyloplast sedimentation. Wm inhibits PI3-kinase, reduces the amount of phosphatidylinositol 3-phosphate in cellular membranes (Jung *et al.*, 2002), and induces vacuole fusion in *zig-1* (Zheng *et al.*, 2014*b*). Seedlings were treated with 33 μM Wm in the vertical microscope stage (see Supplementary Fig. S1 at *JXB* online), and time lapses were collected within 3–8 min of Wm application and after 90 min. Figure 4A shows the orientation of the seedlings throughout the experiment. No obvious differences in amyloplast dynamics were observed in WT seedlings 3–8 min after Wm application (Fig. 4B, 3–8 min) when compared with the untreated control (Fig. 2B). Additionally, amyloplast movements still appeared severely limited in *zig-1* when seedlings were imaged within 8 min of Wm treatment (Fig. 4C, 3–8 min). After 90 min of Wm treatment, WT amyloplasts were still sedimented in the direction of gravity, but the amplitude of saltatory movements seemed reduced (Fig. 4B, 90–93 min). In contrast, 90 min of Wm treatment in *zig-1* resulted in many amyloplasts being found at the bottom of the cell (Fig. 4C, 90–93 min), compared with the untreated *zig-1* (Fig. 2C). Seedlings were then rotated 90° and they were again imaged for 3 min immediately after. Upon reorientation, WT amyloplasts sedimented to the new gravity-associated cell bottom within 3 min (Fig. 4B, rotation). Finally, several amyloplasts in Wm-treated *zig-1* sedimented in the direction of gravity following reorientation and their tracks were more similar to WT (Fig. 4C, rotation). Collectively, these results suggest that Wm treatment, which induces vacuole fusion, is sufficient to release amyloplasts in the endodermis of *zig-1* hypocotyls.

Quantification of amyloplast movements during these experiments indicated that amyloplast saltatory motion in WT was large (up to 20 μm) and showed no obvious bias in direction shortly after Wm treatment (Fig. 4D, 3–8 min). However, Wm treatment for 90 min restricted amyloplast movement in WT to within 10 μm of their initial position (Fig. 4D, 90–93 min). Again, most amyloplasts were able to translocate to the new gravity-associated bottom of the cell following the reorientation (Fig. 4D, rotation). In contrast, Wm treatment of *zig-1* mutants had dramatic effects on amyloplast movement. Initially, *zig-1* amyloplast movement was highly restricted to within 5 μm of their starting position immediately after Wm application (Fig. 4E, 3–8 min) in a similar fashion to untreated seedlings (Fig. 2C). However, after 90 min their range of motion had increased to within 10 μm, and sometimes up to 17 μm from their original position (Fig. 4E, 90–93 min). After rotation, several amyloplasts were able to relocate to the bottom of the cell and move up to 30 μm (Fig. 4E, rotation). These data confirm that altering vacuolar membrane morphology has profound effects on amyloplast movement in *zig-1*.

### Wortmannin treatment of *zig-1* statocytes partially restores gravitropism

**Fig. 5.**
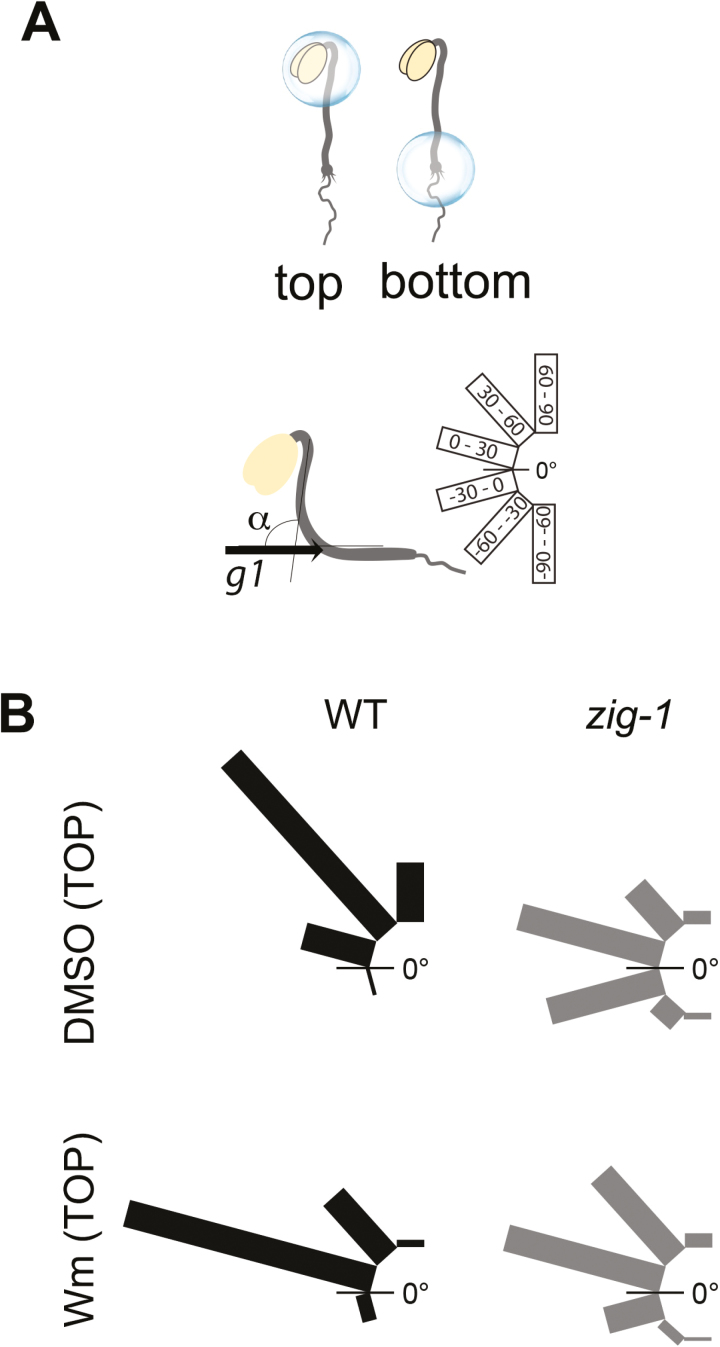
Local Wm application at the top of hypocotyls enhances gravitropism of *zig-1*. Three-day-old seedlings from Col-0 WT and *zig-1* were treated at either the top or the bottom of the hypocotyl with a dot of AGM containing DMSO or 33 μM Wm. Seedlings were then exposed to a 90° rotation, and the angle of hypocotyl curvature was measured after 20 h. (A) Illustrations showing the placement of medium dots containing DMSO or Wm at either the top or the bottom of hypocotyls, the angle of hypocotyl curvature between the first gravity vector (*g*1) and the top of the hypocotyl, and how the angle distributions are represented. (B) Application of Wm at the top of the hypocotyl enhances *zig-1* gravitropism. Shown are the angle distributions for WT and *zig-1* treated with DMSO or Wm at the top of the hypocotyl (*n*=87–112). (This figure is available in color at *JXB* online.)

We have shown above that statocytes in the Arabidopsis hypocotyl may be located just below the hook (Fig. 1). To confirm this, we took advantage of the effect of Wm on *zig-1* vacuoles and amyloplast sedimentation (Fig. 4). Thus, we tested whether local Wm application to either the top or bottom of the hypocotyl was sufficient to restore gravity response in *zig-1*. A small dot of growth medium containing either 1% (v/v) DMSO (control) or 33 μM Wm was placed over the top or the bottom of the hypocotyl. The plates were then rotated 90° from the original gravity vector and the angle of hypocotyl curvature (α) was measured after 20 h (Fig. 5A). Col-0 WT responded to the new gravity vector when DMSO (control) was placed at the top of the hypocotyls, while *zig-1* displayed an agravitropic response, as expected (Fig. 5B). When medium containing Wm was placed at the top of Col-0 hypocotyls, it induced a slight delay in graviresponse as indicated by an increase in the seedlings with an angle between 0 and 30° and a decrease in those between 30 and 60°. However, local application of Wm to the top of *zig-1* hypocotyls resulted in an enhanced response to gravity. *zig-1* mutants treated with DMSO showed a strong agravitropic phenotype, with most seedlings in the –30 to 0° and 0 to 30° categories, while *zig-1* mutants treated with Wm at the top showed an increase in the hypocotyls between 30 and 60° and a decrease in those between –30 and 0° (Fig. 5B). These results indicate that Wm application to the upper hypocotyl partially suppresses the agravitropic phenotype of *zig-1* mutants. In contrast, application of Wm to the bottom of the hypocotyl did not have major effects on gravity response in *zig-1* (see Supplementary Fig. S2). Therefore, only local Wm application to the top of *zig-1* hypocotyls can enhance their gravitropic response.

### Loss of *VTI11* has dramatic consequences for endoplasmic reticulum organization

**Fig. 6.**
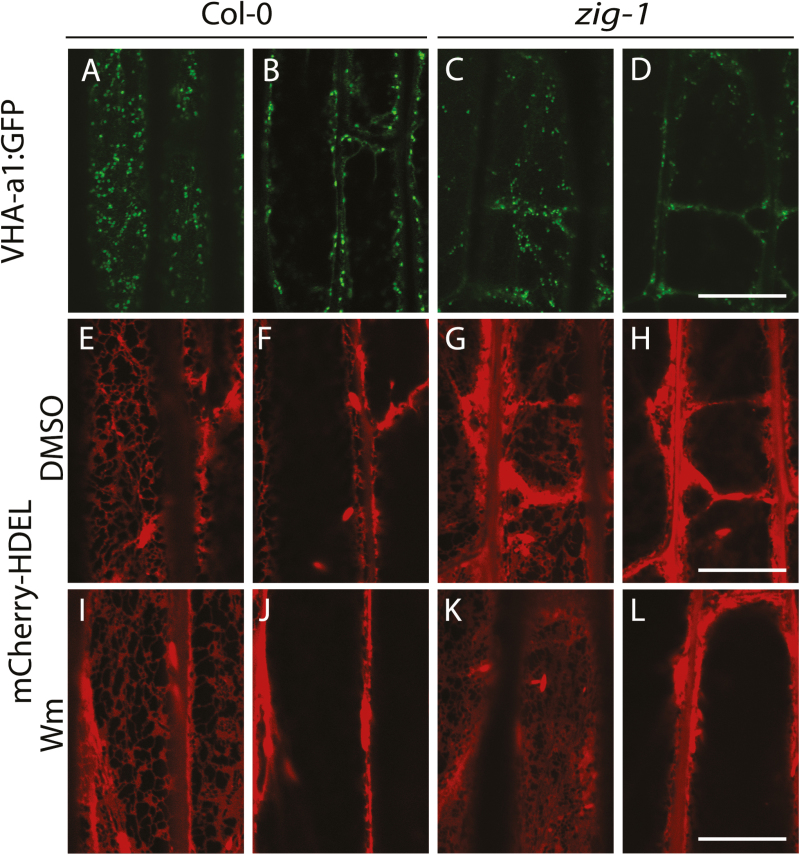
Loss of *VTI11* function results in impaired ER morphology. Hypocotyl cells from 3-d-old seedlings from Col-0 wild-type or *zig-1* expressing subcellular markers were imaged by confocal microscopy. Seedlings were grown in the dark. A cortical section and a medial section of the same cell are shown for each marker. (A–D) Col-0 wild-type or *zig-1* expressing VHA-a1-GFP. (E–L) Dark-grown Col-0 WT or *zig-1* seedlings expressing the ER marker mCherry-HDEL were treated with 1% (v/v) DMSO (E–H) or 33 μM Wm (I–L) for 3 h and then transferred to AGM for 2 h before imaging. Identical microscope settings were used for both genotypes. Scale bar: 20 μm.

The dramatic restriction on amyloplast movement in *zig-1* prompted us to determine if fragmented vacuoles altered the morphology of organelles of the endomembrane system, which could impede their flow. To this end, VHA-a1-GFP, a marker for the *trans*-Golgi network (TGN) (Dettmer *et al.*, 2006), and mCherry-HDEL, a marker for the endoplasmic reticulum (ER) (Nelson *et al.*, 2007), were analysed in the *zig-1* background. Organelle morphology was analysed in hypocotyl cortical cells given the weak fluorescence of these markers in the endodermis. VHA-a1-GFP labeled typical TGN structures in WT and the *zig-1* background (Fig. 6A–D). Not surprisingly, TGN puncta were found both in the cell cortex and in the cytosol that surrounded fragmented vacuoles of *zig-1* (Fig. 6C, D). Therefore, loss of *VTI11* does not appear to have an effect on TGN morphology. The mCherry-HDEL marker showed the canonical ER network organization at the cell cortex and ER bodies in WT (Nelson *et al.*, 2007) (Fig. 6E, F). The ER was found surrounding the small vacuoles of *zig-1*, similar to the other organelles, but two distinct abnormalities could be detected. First, the cortical organization of the ER was altered as there was enrichment of ER membranes in the cortical domains that abutted juxtaposed vacuoles (Fig. 6G). In addition, aggregates of ER were found in the cytosolic domains between juxtaposed vacuoles and these appear to have restrictions in their movement (Fig. 6H and Supplementary Movies S1 and S2). These aggregates of ER could result from accumulation of fusiform bodies at these sites, in addition to aggregated ER membranes, but these are difficult to differentiate at the resolution of a light microscope. These defects indicate that vacuole morphology is important for proper ER organization. To further test this possibility, we determined whether Wm treatment of *zig-1* would restore the cortical ER organization by promoting vacuole fusion. There were no apparent changes to the ER network in WT hypocotyls after treatment with Wm (Fig. 6I, J), although membrane aggregates were sometimes observed. As expected, Wm treatment induced the fusion of vacuoles in *zig-1*, and the enrichment of ER membranes was no longer observed in the cortex after a 2 h washout (Fig. 6K, L). These results overall indicate that vacuole morphology defects in *zig-1* do not alter the morphology of the TGN, but it does have a dramatic effect on the organization of the ER.

### Vacuoles and amyloplasts associate by cytoskeleton-independent mechanisms

**Fig. 7.**
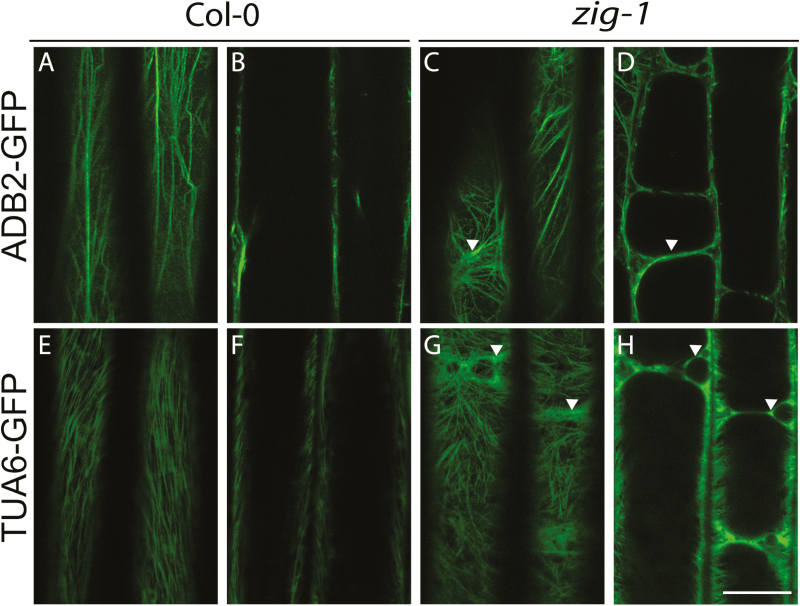
The cytoskeleton organization at the cortex is not affected in *zig-1*. Four-day-old seedlings from Col-0 WT (A, B, E, F) or *zig-1* (C, D, G, H) expressing ABD2-GFP to label F-actin (A–D) or TUA6-GFP to label microtubules (E–H) were imaged by confocal microscopy. Both cortical sections (A, C, E, G) and medial sections (B, D, F, H) for a representative cell are shown. Seedlings were grown in the dark. Arrowheads indicate zones with cytoskeleton enrichment that are found above juxtaposing vacuoles. Scale bar: 20 μm. (This figure is available in color at *JXB* online.)

F-actin is involved in amyloplast saltatory movements in wild-type (Saito *et al.*, 2005; Yamamoto and Kiss, 2002), and it may function as a negative regulator of sedimentation (Nakamura *et al.*, 2011), but its specific role in the gravity response is unknown (Blancaflor, 2013). Because amyloplasts appear trapped between fragmented vacuoles in *zig-1* (Fig. 1) and are relatively static (Fig. 2), it was important to investigate if F-actin or microtubules were involved in restricting their movement. The cytoskeleton was visualized using the F-actin marker ABD2-GFP (Wang *et al.*, 2004) or microtubule (MT) marker TUA6-GFP (Ueda *et al.*, 1999) in cortical and medial sections of epidermal cells (Fig. 7). F-actin cables were observed at the cortex in *zig-1* (Fig. 7C) similar to the WT (Fig. 7A), except that the F-actin network surrounds the fragmented vacuoles in *zig-1* (Fig. 7C). We conclude that the fragmented vacuoles in *zig-1* do not impede F-actin polymerization or cortical arrangement. Similarly, microtubules were detected in the cell cortex in WT (Fig. 7E) and *zig-1* (Fig. 7G). However, dense MT arrays were also observed close to juxtaposing vacuoles (Fig. 7H). Overall, the lack of a central vacuole in *zig-1* does not appear to cause aggregates or abnormalities in the composition of the cytoskeletal network, except that both polymers are found surrounding the *zig-1* vacuoles.

**Fig. 8.**
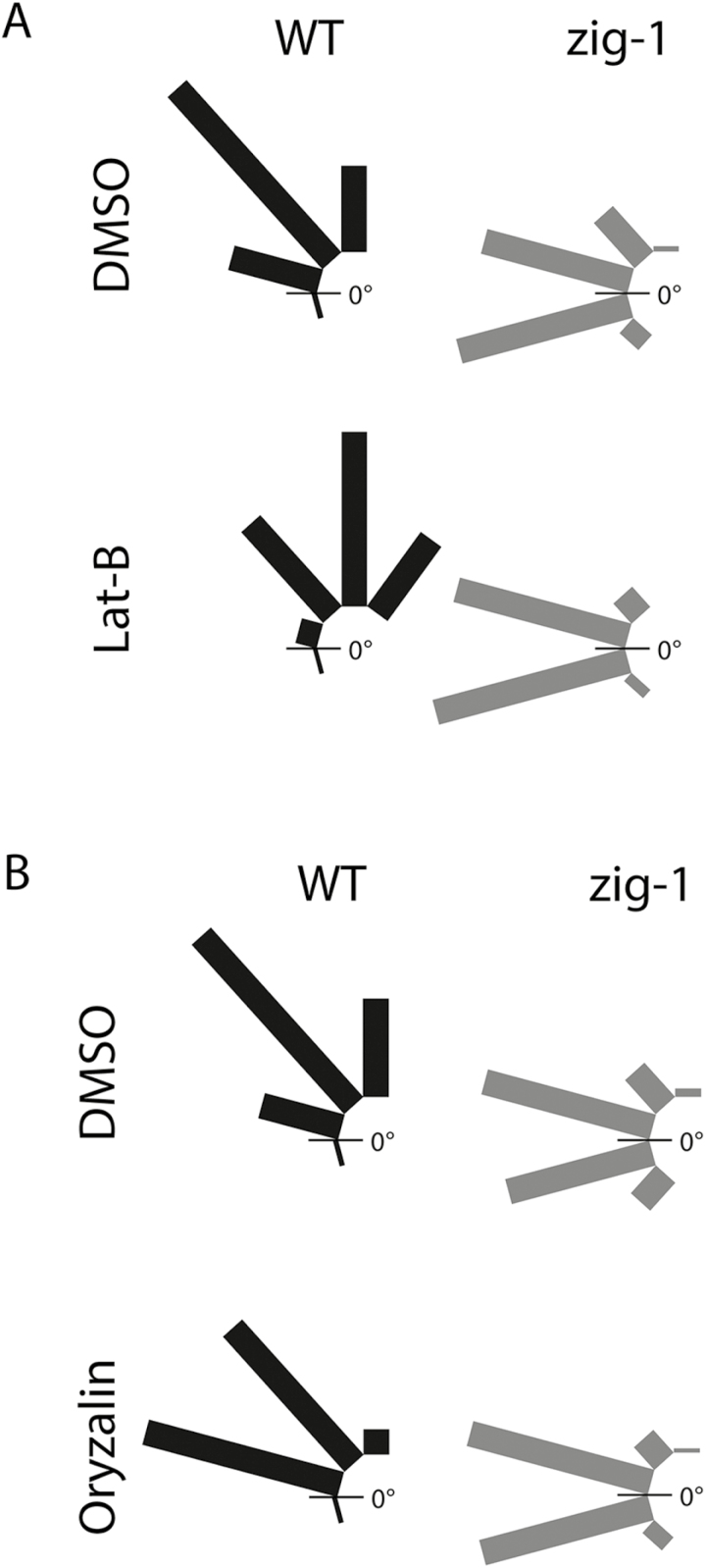
Neither lantrunculin B (Lat B) nor oryzalin treatment suppresses the agravitropic phenotype of *zig-1*. Gravitropism assay with Col-0 WT and *zig-1* in the presence of cytoskeleton inhibitors. Seedlings were grown vertically for 3 d in the dark, and then transferred to AGM plates containing 1% (v/v) DMSO (A, B), 2 μM Lat-B (A) or 20 μM oryzalin (B). Plates were returned to the dark and oriented at 90° from the original gravity vector (*g*1). After 20 h the angle of hypocotyl curvature was measured between *g*1 and the top of the hypocotyl. The distribution of seedlings in each degree class is indicated. *n*=77–100.

We conducted gravitropic assays in the presence and absence of the cytoskeleton inhibitors lantrunculin B (Lat-B) or oryzalin to determine if the cytoskeleton contributed to the static behavior of *zig-1* amyloplasts. Lat-B induced an increase in the response to gravity in WT including an increase in the hypocotyls between 60 and 90° and hypocotyl curvatures of more than 90° in 22.1% of seedlings (Fig. 8A). No changes were observed in *zig-1*, with or without Lat-B, as most *zig-1* hypocotyls were found in two categories, –30 to 0° and 0 to 30°, in both treatments (Fig. 8A). The ABD2-GFP marker was included in these experiments as a control to show the effectiveness of the Lat-B treatment (see Supplementary Fig. S3A). A possible role of MT in tethering of amyloplasts was also tested (Fig. 8B). There was a delay in the response of the oryzalin-treated seedlings, with an increase in the proportion of seedlings in the 0–30° category and a decrease in the 60–90° category. In the case of *zig-1*, both treatments resulted in very similar angle distributions, with almost all seedlings bending to an angle between –30 and 30°. Therefore, treatment with oryzalin does not enhance graviresponse of *zig-1*. Again, the TUA6-GFP marker was used to confirm the effectiveness of the oryzalin treatment (Supplementary Fig. S3C).

That neither Lat-B nor oryzalin treatments suppressed the agravitropic phenotype of *zig-1* suggested that neither actin nor MT depolymerization was sufficient to release amyloplasts from the static inter-vacuolar junctions. To confirm this, amyloplast sedimentation was visualized after inhibitor treatment in the endodermis of dark-grown hypocotyls (see Supplementary Fig. S3B). In this experiment, seedlings were incubated overnight in the presence of DMSO, 2 μM Lat-B or 20 μM oryzalin to depolymerize the cytoskeleton. Seedlings were then incubated vertically for 10 min, imaged, incubated vertically again for 10 min but upside down (180° rotation), and then imaged again. Wild-type amyloplasts sedimented to the new bottom of the cell after the 180° rotation regardless of chemical pretreatment. In contrast, *zig-1* amyloplasts did not sediment in either of the chemical treatments as shown by their static localization before and after reorientation (Supplementary Fig. S3B). Since depolymerization of F-actin or MT does not enhance gravity response in *zig-1*, nor does it restore amyloplast sedimentation, these results overall indicate that actin filaments or microtubules are not contributing to the immobile nature of amyloplasts by serving as a physical tether connecting amyloplasts and tonoplast. Therefore, the static nature of *zig-1* amyloplasts is not explained by physical associations with the cytoskeleton.

### Wm enhances graviresponse of *vti11* alleles through dynamic TVS formation

**Fig. 9.**
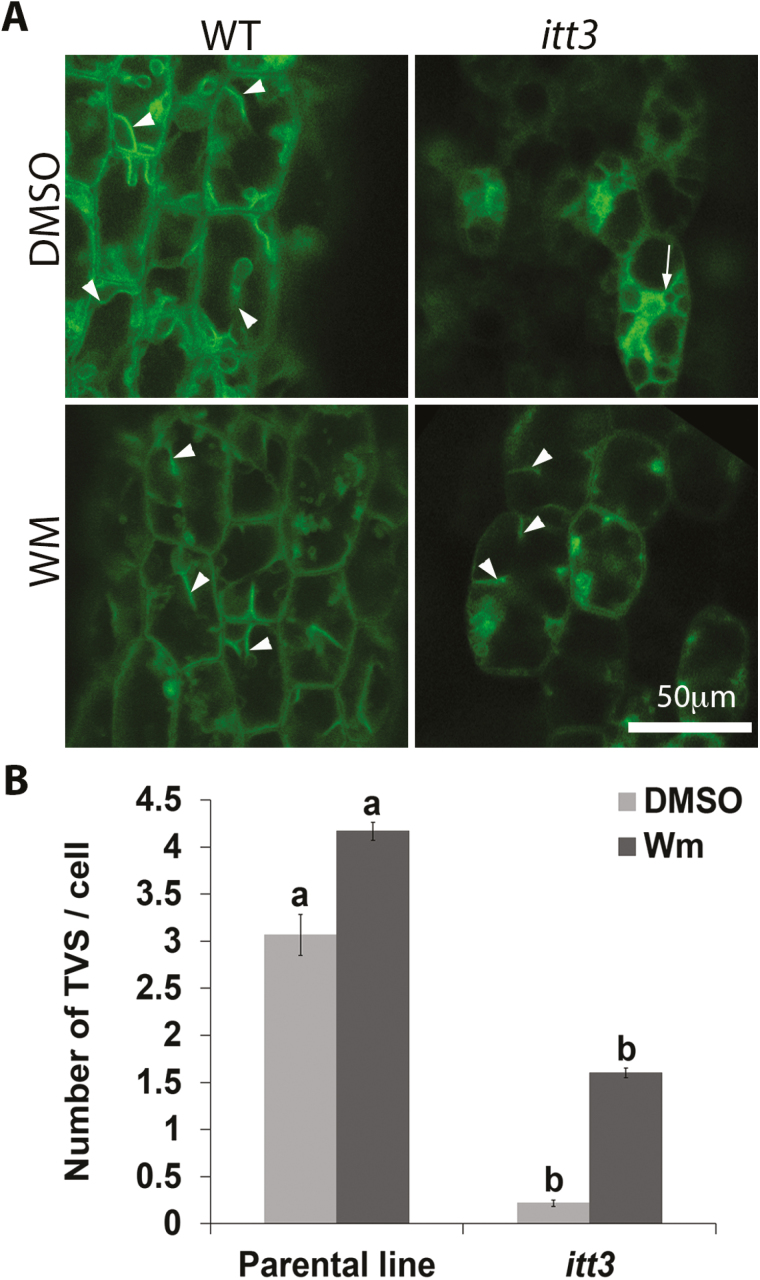
Wm treatment induces the formation of transvacuolar strands (TVSs). (A) Thick optical sections were captured for TVS quantification at low magnification. Example images used from WT and *itt3* hypocotyls are shown for both DMSO and Wm treatments. The types of TVS that were counted are indicated with arrowheads. Note that membranes that fully surrounded a vacuole in *itt3* were not included (arrows). (B) Average number of TVSs per cell in parental and *itt3*. *n*=60 cells total from four seedlings per genotype. Mean values are statistically significant (Student’s *t* test, two tailed, *P*≤0.05) if letters are different.

We have demonstrated that immobile amyloplasts in *zig-1* hypocotyls increase dynamic movements after Wm treatments. We then quantified transvacuolar strands (TVSs) in hypocotyls of WT and *itt3* before and after Wm treatment (Fig. 9A) to test whether this enhanced motion is due to vacuolar membrane remodeling through changes in TVS formation. The parental line showed 3.07 ± 0.22 TVSs per cell in hypocotyl epidermis, while *itt3* mutants had only 0.22 ± 0.04 TVSs when DMSO treatments were used (Fig. 9B). The number of TVSs increased to 4.17 ± 0.10 in the parental line and to 1.60 ± 0.05 for *itt3* when seedlings were treated with Wm for 2 h. The significant increase in the number of TVSs after Wm treatment for both the parental line and *itt3* suggests that Wm not only promotes vacuole fusion, but has a positive effect on vacuole membrane dynamics as well.

## Discussion

Here, we used live-cell imaging to analyse the dynamics of amyloplast sedimentation in Arabidopsis hypocotyls from whole seedlings. Previous studies have utilized cuttings from inflorescence stems (Morita *et al.*, 2002; Saito *et al.*, 2005; Toyota *et al.*, 2013*b*). Our microscope set-up allowed us to image endodermis through the almost transparent cortex of etiolated hypocotyls using a fluorescence microscope. A very small amount of Immersol allowed us to use a water immersion objective on the vertical stage, which provided optimal resolution. This unique set-up allowed us to track amyloplast sedimentation in real time and in whole plants without the concerns of plant injury. The system is amenable for cell biology analysis, including treatment with chemical inhibitors, and long-term time-lapse experiments. Furthermore, this system can overcome the challenges of imaging using stem cuttings, which have been shown to affect the vacuole morphology of certain mutants (Hashiguchi *et al.*, 2014). One caveat of our system is the need to illuminate the seedlings during image acquisition, which we have addressed with a green filter to minimize phototropic responses. Green light, however, has been shown to enhance hypocotyl growth and inhibit blue and red phototropic responses in dark-grown seedlings (Folta, 2004; Folta and Maruhnich, 2007; McCoshum and Kiss, 2011). Therefore, experiments in the vertical stage must be interpreted with caution as they may be influenced by effects of the green light on seedling growth.

### Restriction of amyloplast sedimentation in *zig-1* is dependent on its vacuole phenotype

A role for the vacuole in gravity perception or response is well documented (Morita *et al.*, 2002; Yano *et al.*, 2003; Saito *et al.*, 2005). The *VTI11* loss of function mutant *sgr4*/*zig-1* was originally identified for its agravitropic phenotype in shoots (Yamauchi *et al.*, 1997; Kato *et al.*, 2002), and it was later shown to display abnormal vacuole morphology and reduced vacuole fusion (Zheng *et al.*, 2014*b*). We used *zig-1* and a second allele, *itt3*, as tools to interrogate the role of vacuole dynamics on gravitropism in Arabidopsis hypocotyls. As expected, WT endodermal cells from hypocotyl statocytes show a large central vacuole and sedimented amyloplasts that appeared surrounded by tonoplast membrane. This organization is very similar to that found in inflorescence stems (Morita *et al.*, 2002). In contrast, and consistent with other tissues (Zheng *et al.*, 2014*a*,b), smaller unfused vacuoles were detected in endodermal statocytes from *zig-1* mutants, where the amyloplasts were found distributed almost randomly throughout the cell. In these cells, amyloplasts appeared trapped at the junction of juxtaposing vacuoles, and this organization seems to prevent amyloplast movement in *zig-1*, both before and after gravistimulation, as depicted by time-lapse experiments. Surprisingly, conditions of hypergravity up to 10 *g* were not sufficient to force amyloplasts to sediment in these cells. It is unlikely that the fragmented vacuoles alone would be sufficient to prevent the movement of these organelles simply by creating physical obstacles to their movements along the cytosolic space. Therefore, we propose that a physical tether exists between the vacuole and amyloplasts that is of sufficient strength to resist a 10 *g* force. In fact in the case of *zig-1*, each amyloplast seems attached to multiple vacuoles, which perhaps generates a strong physical tethering structure to resist this hypergravity pull. In contrast, hypergravity is known to increase amyloplast sedimentation and gravitropic curvature of other agravitropic mutants such as *sgr2*, *sgr9*, and *pgm* (Toyota *et al.*, 2013*b*), but none of these mutants displays a fragmented vacuole phenotype. Amyloplast sedimentation has been shown to occur via transvacuolar strands (Morita *et al.*, 2002; Saito *et al.*, 2005), and therefore we asked whether the fragmented vacuole phenotype of *zig-1* could impair TVS formation. In fact, *zig-1* mutants formed very few TVS structures in epidermal cells when compared with the WT, which could contribute to the reduced sedimentation of amyloplasts inside their statocytes. This possibility is consistent with the phenotype of the *sgr2* mutant, which displays fewer TVSs than WT and is agravitropic (Morita *et al.*, 2002; Saito *et al.*, 2005).

Treatment with Wm induces fusion and restores the vacuole morphology of *zig-1* (Zheng *et al.*, 2014*a*,b). We used this assay to test whether the vacuole morphology phenotype of *zig-1* was necessary for their lack of amyloplast sedimentation and the role of vacuole morphology on this movement. As expected, Wm treatment increased amyloplast sedimentation in *zig-1*. The Wm-induced vacuole fusion may have this effect due to the following: (i) vacuole fusion could disrupt the putative tethers between amyloplasts and the vacuolar membrane proposed above and therefore increase mobility of these organelles; (ii) vacuole fusion could restore the ability to form TVSs, which could become conduits for amyloplast sedimentation; (iii) restoring the single vacuole phenotype could remove physical obstacles formed by the vacuoles themselves. We indeed confirmed that Wm-induced vacuole fusion increases the formation of TVSs in epidermal cells. However, TVSs alone are probably not sufficient because *sgr2* mutants, which do not form TVSs in stems, were able to reposition amyloplasts along a hypergravity vector (Toyota *et al.*, 2013*b*). Future research is needed to address the role of a putative tethering system to determine if all three possibilities play a role in this response. The alternate possibility that Wm is actually affecting gravitropism through effects on cellular events unrelated to vacuolar function seems less likely as Wm action on gravity response is not seen in WT plants where these other sites of action would presumably still be present.

### Shoot statocytes comprise young endodermal cells near the top of the hypocotyl

The endodermal layer of the hypocotyl has been identified as the graviperceptive tissue in shoots (Fukaki *et al.*, 1998). Visualization of endodermal cells in the medial region of the hypocotyl of etiolated seedlings indicated that most cells did not show the expected sedimented amyloplasts, and instead these organelles were in constant motion from cytoplasmic streaming. Only the smaller cells in the upper hypocotyl of an average length of 70 μm or less showed sedimented amyloplasts that were consistent with graviperception. To confirm this result, we took advantage of the phenotype of *zig-1* and Wm treatment. This provided a unique opportunity to test whether amyloplast sedimentation in *zig-1* at the top or the bottom of the hypocotyl was associated with restoration of gravitropism. Adding Wm at the top of the hypocotyl enhanced gravitropism in these mutants, but the treatment at the bottom did not. Therefore, only Wm treatment at the top is sufficient to restore the gravity response. This effect is correlated with an increase in amyloplast sedimentation as shown by organelle tracking experiments. Altogether, these results indicate that the statocytes in etiolated hypocotyls are localized to the young endodermal cells at the top of the hypocotyl. These results are consistent with the top ~4 cm region of the inflorescence stem being sufficient for graviresponse in mature plants (Fukaki *et al.*, 1996*a*) and the finding that displacement of amyloplasts at the base of the inflorescence stem does not result in inflorescence bending (Weise *et al.*, 2000).

### The cytoskeleton is not involved in amyloplast tethering to *zig-1* vacuoles

A role for actin during shoot gravitropic perception remains unclear (Yamamoto and Kiss, 2002; Yamamoto *et al.*, 2002; Palmieri and Kiss, 2005; Saito *et al.*, 2005; Blancaflor, 2013). The inability of amyloplasts to sediment in *zig-1* could be due to the fragmented vacuoles being obstacles for movement. However the fact that hypergravity is not sufficient to release them indicates that there may be physical tethers between the amyloplasts and the vacuole. We tested whether such tethers could be formed in part by cytoskeletal structures and, therefore, if they could be disrupted with inhibitors that depolymerize MT and actin microfilaments. However, neither Lat-B nor oryzalin treatment was sufficient to overcome the block in sedimentation. This is consistent with previous reports indicating that the tight association between vacuole and amyloplasts was not disrupted by lantrunculin B in wild-type inflorescences (Saito *et al.*, 2005). However, these results are not consistent with experiments in flowering snapdragon shoots, in which depolymerization of actin microfilaments with Lat-B released amyloplasts (Friedman *et al.*, 2003). We conclude that the association between amyloplasts and the vacuole may occur either directly or indirectly but does not seem to involve the cytoskeleton. Contact sites between the vacuole and other organelles and association between those and amyloplasts may be responsible for the tethering that we have observed. Contact sites between plant subcellular organelles have been identified by electron microscopy or laser trapping, including between ER and plasma membrane (summarized in Staehelin, 1997), ER and endosomes (Stefano *et al.*, 2015), and ER and Golgi (Sparkes *et al.*, 2009). The vacuole has also been shown in tight association with the cytoskeleton (Scheuring *et al.*, 2016) and the ER (Staehelin, 1997), and the latter may provide an anchoring system that may be important to regulate amyloplast sedimentation. Future analysis by electron microscopy in shoot endodermal cells may provide further support for these contact sites. The dramatic effects on ER morphology in the *zig-1* mutant indicates that ER–vacuole interactions are critical for organelle structure. Given that no major changes in organelle morphology or distribution were observed for the TGN, we conclude that the vacuole is unlikely to form physical attachments with these organelles as it does for amyloplasts.

## Supplementary data

Supplementary data are available at *JXB* online.

Fig. S1. Microscope set-up for live-cell imaging with a vertical stage and a sample chamber.

Fig. S2. Local Wm application at the bottom of hypocotyls does not enhance gravitropism of *zig-1*.

Fig. S3. Lat-B and oryzalin depolymerize cytoskeleton, but do not free amyloplasts in the *zig-1* background.

Movie S1. Time lapse of the ER marker mCherry-HDEL in dark-grown Col-0 WT.

Movie S2. Time lapse of the ER marker mCherry-HDEL in dark-grown *zig-1*.

Supplementary Data
