# Supplementary material for: Wortmannin-induced vacuole fusion enhances amyloplast dynamics in Arabidopsis zigzag1 hypocotyls
Source: J Exp Bot. 2016 Nov 5;67(22):6459–72. doi: 10.1093/jxb/erw418 (PMC5181587; doi:10.1093/jxb/erw418)
Supplement: Supplementary Data [file supp_67_22_6459__index.html]

Wortmannin-induced vacuole fusion enhances amyloplast dynamics in Arabidopsis zigzag1 hypocotyls — Wortmannin-induced vacuole fusion enhances amyloplast dynamics in Arabidopsis zigzag1 hypocotyls — Supplementary Data 

# Wortmannin-induced vacuole fusion enhances amyloplast dynamics in Arabidopsis *zigzag1* hypocotyls

## Supplementary Data

Data files

- supplementary\_figures\_S1\_S3.pdf - Supplementary Data
- supplementary\_movie\_S1.avi - Supplementary Data
- supplementary\_movie\_S2.avi - Supplementary Data
