# Supplementary material for: Wortmannin-induced vacuole fusion enhances amyloplast dynamics in Arabidopsis zigzag1 hypocotyls
Source: J Exp Bot. 2016 Nov 5;67(22):6459–72. doi: 10.1093/jxb/erw418 (PMC5181587; doi:10.1093/jxb/erw418)
Supplement: Supplementary Data [file supp_erw418_supplementary_figures_S1_S3.pdf]

Wortmannin-induced vacuole fusion enhances amyloplast dynamics in Arabidopsis *zigzag1* hypocotyls and implicates tonoplast in the gravitropic response

Ashley Ann Alvarez, Sang Won Han, Masatsugu Toyota, Carla Brillada, Jiameng Zheng, Simon Gilroy, and Marcela Rojas-Pierce

Supplemental Figures

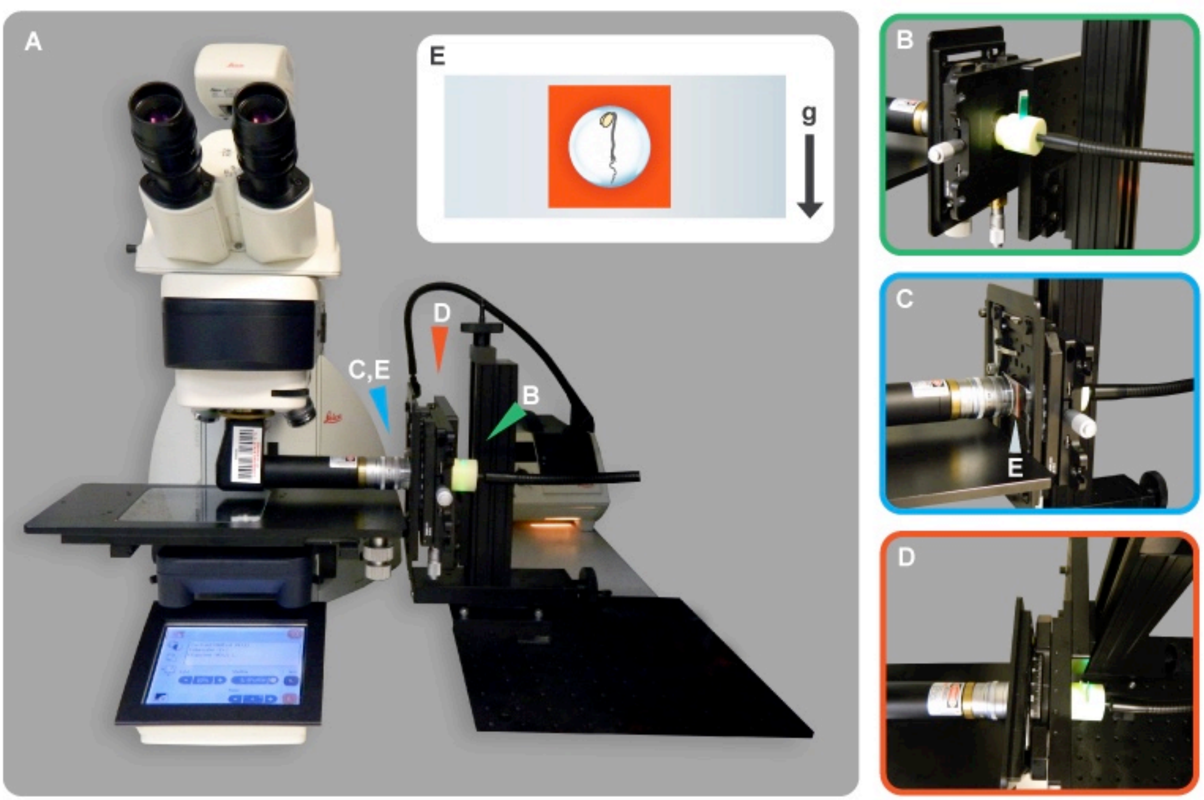

Figure S1. Live-cell imaging with a vertical stage and a sample chamber.
A) Overview of the Leica compound microscope with 90° InverterScope objective inverter and vertical stage.
B) Transmitted light illumination was accomplished with a 3-D printed adaptor to fit a light guide inside the rotating stage. This adapter includes a slit for placement of a green filter. A condenser lens was placed inside the rotating stage between the sample and the light adapter.
C) Position of the 40x water objective and sample.
D) Vertical rotating stage.
E) Samples were mounted on slides by gluing the seedling with medical adhesive to the slide surface. A very small amount of Immersol 518F (Zeiss) was used between the coverslip and the objective as immersion liquid. Immersol has high viscosity which reduces dripping on the vertical stage with a similar refractive index as water. The optimal working distance for the

objective was provided by a 1.0 mm thick silicon isolator between the slide (specimen) and the coverslip. This was sealed with a coverslip to create a leak-proof chamber for long-term imaging.

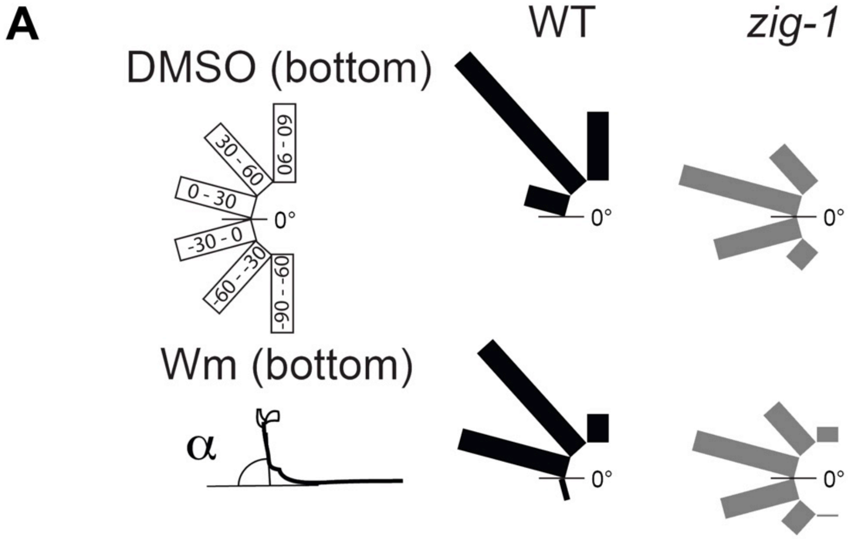

Figure S2. Local Wm application at the bottom of hypocotyls does not enhance gravitropism of *zig-1*.
Dark-grown seedlings of Col-0 wt and *zig-1* were grown vertically on AGM for 3 days. A dot of AGM media containing either 1% v/v DMSO or 33 μM Wm was then transferred to the bottom of the hypocotyl and plates were returned to the dark 90° from the original gravity vector (*g*1). After 20 hours the angle of hypocotyl curvature (α) was measured between g1 and the top of the hypocotyl. Shown is the percentage distribution of seedlings in each degree class (*n*=87-112).

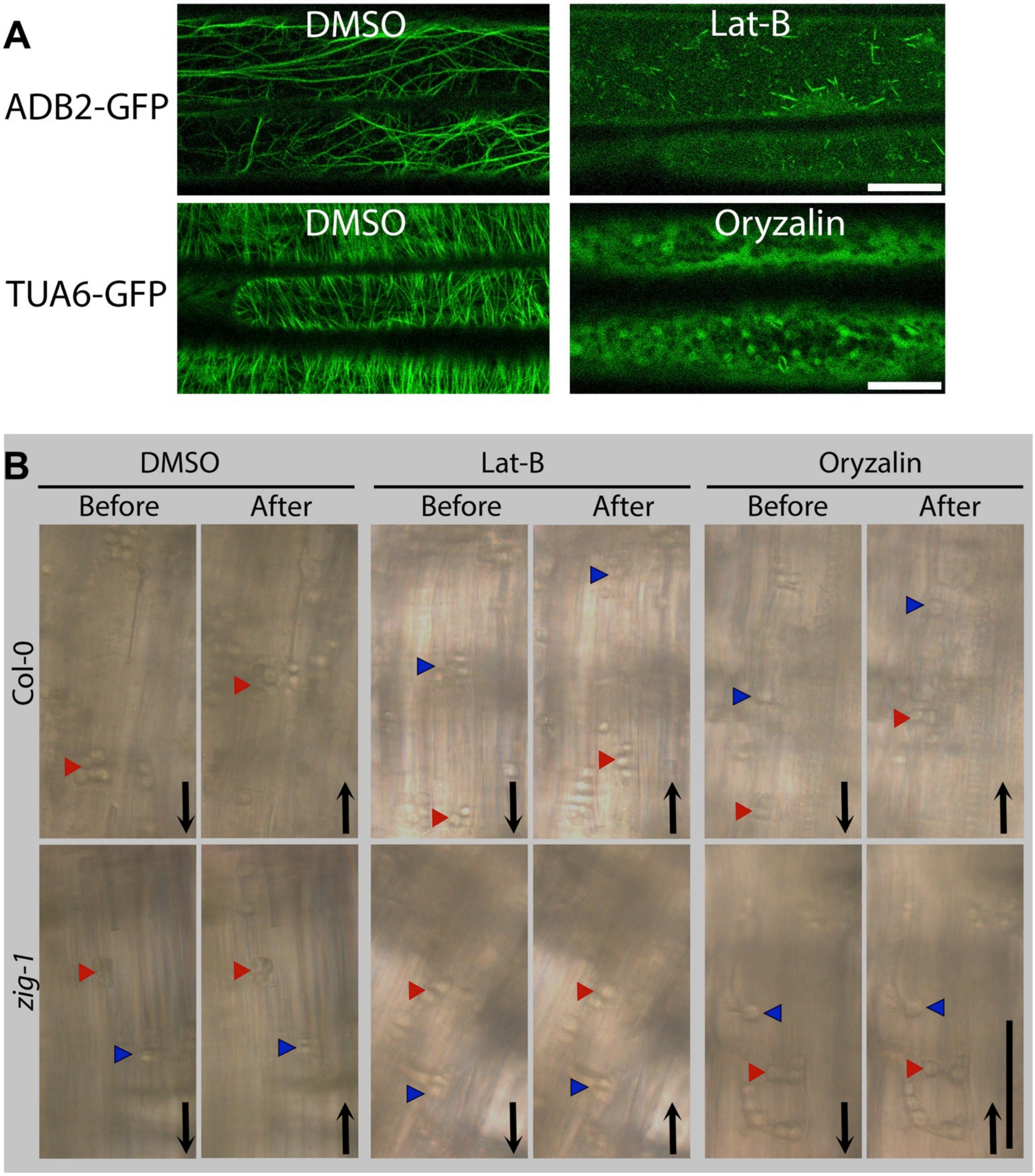

Figure S3. Lat-B and Oryzalin depolymerize cytoskeleton, but do not free amyloplasts in the *zig-1* background.

(A) The cytoskeleton was visualized using ABD2-GFP and TUA6-GFP as controls for the Lat-B and Oryzalin treatments in Figure 7A. Images of the hypocotyl epidermis were collected on a confocal microscope with a 40x water objective. Scale bar = 20 μm.

(B) Amyloplast sedimentation was tested in 4-day-old dark-grown WT or *zig-1* seedlings after treatment with DMSO (control), 2 μM Lat-B or 20 μM Oryzalin. Seedlings were treated for 15-20 h in each chemical. Then, seedlings were mounted on slides and incubated vertically for 10 minutes before being imaged horizontally on a Leica compound microscope (Before).

The slide was then removed from the stage, oriented vertically but up-side down from the original orientation and incubated for 10 min (180° reorientation). Seedlings were then imaged immediately on the horizontal stage (After). Arrowheads of the same color depict the position of groups of amyloplasts before and after the 180° re-orientation. Scale bar = 50 μm

Supplementary Movie Legends

Supplemental Movie 1. Time lapse of the ER marker mCherry-HDEL in dark-grown Col-0 WT.

Hypocotyl cells from 3-day-old Col-0 seedlings expressing mCherry-HDEL were imaged by confocal microscopy. Images were captured every 5 seconds.

Supplemental Movie 2. Time lapse of the ER marker mCherry-HDEL in dark-grown *zig-1*.

Hypocotyl cells from 3-day-old *zig-1* seedlings expressing mCherry-HDEL were imaged by confocal microscopy. Images were captured every 5 seconds.
